# Unveiling the Pathogenic Role of Novel *CPLANE1* Compound Heterozygous Variants in Joubert Syndrome: Insights Into mRNA Stability and NMD Pathway

**DOI:** 10.1111/jcmm.70484

**Published:** 2025-03-12

**Authors:** Zhidan Hong, Sheng Xiang, Zhiying Chen, Xueping Qiu, Li Zhang, Ling Ma, Mei Wang

**Affiliations:** ^1^ Center for Reproductive Medicine Zhongnan Hospital of Wuhan University Wuhan Hubei People's Republic of China; ^2^ Clinical Medicine Research Center of Prenatal Diagnosis and Birth Health in Hubei Province Wuhan Hubei People's Republic of China; ^3^ Wuhan Clinical Research Center for Reproductive Science and Birth Health Wuhan Hubei People's Republic of China; ^4^ Department of Obstetrics and Gynecology Huangmei County People's Hospital Huanggang China; ^5^ Center for Gene Diagnosis, Department of Laboratory Medicine Zhongnan Hospital of Wuhan University Wuhan China; ^6^ Maternal and Child Health Hospital of Hubei Province Wuhan Hubei People's Republic of China

**Keywords:** alternative splicing, *CPLANE1*, Joubert syndrome, nonsense‐mediated mRNA decay, novel compound heterozygous variants

## Abstract

Joubert syndrome (JS) is a rare neurodevelopmental disorder associated with mutations in genes involved in ciliary function. Germline variants in *CPLANE1* have been implicated in JS. In this study, we investigated a family with three adverse pregnancies characterised by fetal malformations consistent with JS. Whole‐exome sequencing (WES) identified compound heterozygous variants in *CPLANE1*: c.8893C>T (p.Gln2965*) and c.203C>T (p.Thr68Ile). Sanger sequencing confirmed the variants in the family. Bioinformatics analysis predicted that the c.203C>T variant affects mRNA splicing and protein function. Functional studies using PBMCs demonstrated that the c.203C>T variant causes exon 3 skipping, resulting in a frameshift and premature termination codon, leading to potential nonsense‐mediated mRNA degradation (NMD). The mRNA transcription and translation inhibition experiment, by treatment with actinomycin D and puromycin, indicated that the c.203C>T variant leads to accelerated mRNA degradation. Notably, the inhibition of SMG1, a key marker of the NMD pathway, partially rescued mRNA expression in mutated cells, providing further evidence of NMD activation. Based on these findings and ACMG guidelines, the c.203C>T variant was reclassified from a variant of uncertain significance (VUS) to likely pathogenic. This is the first report of novel *CPLANE1* compound heterozygous variants contributing to JS in this family. Our study expands the known pathogenic variant spectrum of *CPLANE1* in JS and provides new insights into the molecular mechanisms of this ciliopathy.

## 1 Introduction

Joubert syndrome (JS) is a rare autosomal recessive ciliopathy, with an estimated prevalence of 1 in 80,000 to 100,000 live births [1]. The hallmark of JS is the molar tooth sign (MTS), a distinctive brain malformation resulting from structural defects in the cerebellar vermis and brainstem [2]. Clinically, JS presents with a spectrum of neurological and systemic symptoms, including developmental delays, hypotonia, abnormal breathing patterns, and ataxia. In severe cases, progressive visual loss, renal failure, and significant motor and cognitive impairments can occur, severely impacting quality of life and long‐term prognosis [3].

Genetically, JS is highly heterogeneous, with over 35 causative genes identified to date, including *AHI1*, *TMEM67*, and *CEP290* [4]. Among these, *CPLANE1*, a gene encoding a protein essential for ciliary transition zone assembly, has been implicated in more severe forms of JS [5, 6]. Despite recent advances, the full spectrum of *CPLANE1*‐associated variants and their contributions to JS pathogenesis remain incompletely understood. Identifying novel variants in this gene is crucial for refining genotype–phenotype correlations and improving the precision of genetic counselling and prenatal diagnosis for affected families.

In this study, we report the identification of novel compound heterozygous variants in *CPLANE1* in a JS family, expanding the mutational spectrum of this gene and offering new insights into its role in the clinical manifestation of JS. Through comprehensive clinical assessment and whole exome sequencing (WES), followed by rigorous bioinformatic analysis and in vivo/in vitro validation, we demonstrate the functional relevance of these variants. In vivo validation assessed variant segregation within the family, while in vitro studies evaluated their effects on alternative splicing and protein function, further elucidating their pathogenicity.

This study highlights the crucial role of integrating detailed clinical evidence, WES, bioinformatic analysis, and in vitro experiments to investigate the impact of variants on mRNA splicing and protein translation. Furthermore, we delve into the involvement of mRNA degradation pathways, particularly NMD, in variant‐induced dysregulation. This work demonstrates how novel variants can be successfully reclassified from Variant of Uncertain Significance (VUS) to Likely Pathogenic (LP), providing essential evidence for clinical genetic counselling and advancing our understanding of rare genetic disorders like Joubert syndrome.

## 2 Materials and Methods

### 2.1 Clinical Data Collection and Ethical Approval

A family with a proband diagnosed with JS who presented to the Center for Reproductive Medicine, Zhongnan Hospital of Wuhan University in January 2024 due to three pregnancies affected by fetal abnormalities was selected for this study. A detailed pedigree was constructed based on the family's reproductive history (Figure 3A). The proband's mother (II‐3), a 37‐year‐old healthy woman, had no history of consanguineous marriage, genetic disorders, or occupational exposure to harmful substances. Her menstrual cycles were regular, with a 35‐day cycle, 6 days of bleeding, normal flow, and no dysmenorrhea.

**FIGURE 1:**
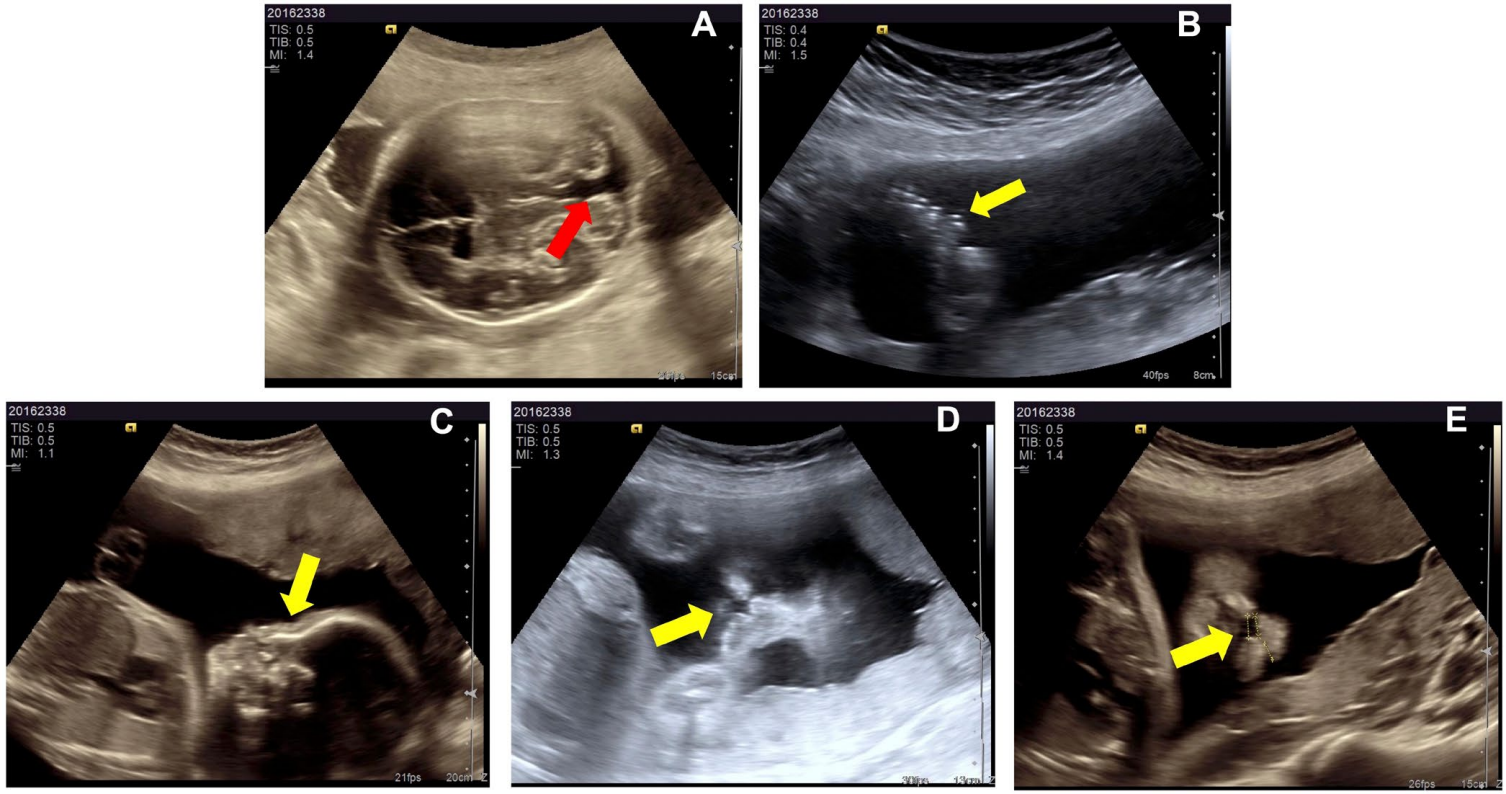
Ultrasonography examination of the fetus at 26 weeks of gestation from the second adverse pregnancy. (A) Absence of the cerebellar vermis, with continuity between the cerebellomedullary cistern and the fourth ventricle (red arrow). (B) Polydactyly (yellow arrow). (C) Flat nasal bridge (yellow arrow). (D) Cleft lip (yellow arrow). (E) A discontinuity of approximately 0.65 cm in the echogenic continuity of the upper lip, with a 0.3 cm discontinuity in the echogenicity of the alveolar arch on the right side (yellow arrow). Ultrasound images taken at 26 weeks of gestation.

The first pregnancy in 2014 was terminated at 16 weeks following an ultrasound, which revealed cerebellar vermis hypoplasia, a cystic structure in the posterior fossa, possible midline cleft lip, bilateral cleft lip and palate, possible arachnoid cysts adjacent to the midline, cavum veli interpositi, and bilateral periventricular haemorrhage. The couple opted for a termination of pregnancy (TOP). In the second pregnancy in 2016, ultrasonography examination at 26 weeks of gestation showed possible syndactyly and polydactyly of both hands and feet, cerebellar vermis agenesis, right‐sided cleft lip, and possible alveolar arch and cleft palate (Figure 1). After thorough consultation regarding the prognosis, the couple voluntarily elected to terminate the pregnancy and consented to genetic testing of the fetus to determine the aetiology.

During the third pregnancy in 2021, an ultrasound examination at a local hospital at 17 weeks of gestation indicated fetal abnormalities. A 0.17 cm continuous midline defect was observed on the upper lip, with no normal nasal echo visible. A nose‐like echo of 0.55 × 0.35 cm was noted above the upper lip. The lateral ventricles measured 1.0 cm, and the cerebellum appeared malformed. Additionally, increased echogenicity of the bowel was observed. After thorough consultation regarding the prognosis, the couple voluntarily elected to terminate the pregnancy and consented to genetic and pathological testing of the fetus to determine the aetiology.

This study was approved by the Ethics Committee of Zhongnan hospital of Wuhan university (Approval No. [2,023,068 K]). Written informed consent for clinical research was obtained from all participating family members.

### 2.2 Fetal Autopsy Examination

A systematic autopsy of the proband (fetus from the third pregnancy) was performed, with all organs examined according to standard anatomical protocols. Tissue samples were collected from each organ following routine procedures. The specific steps of the examination adhered to the guidelines outlined in the *Clinical Technical Operation Specifications (Pathology Volume)* and the *Practical Manual of Fetal Pathology*.

### 2.3 Genomic DNA Extraction From the Proband and Family Members

Genomic DNA was extracted from the proband and family members using EDTA anticoagulant blood collection tubes (Batch No.: 0080230401, Jiangsu Kangjian Medical Supplies Co. Ltd.). Muscle tissue (2 g) was collected from the proband, while 2 mL of peripheral venous blood was collected from both the father and mother. DNA extraction was performed using the column‐based TIANamp Genomic DNA Kit (Batch No. U8709, TIANGEN Biotech Co. Ltd., Beijing, China), following the manufacturer's protocol.

### 2.4 Whole Exome Sequencing and Candidate Pathogenic Variant Screening

WES was conducted to detect genetic variants in the proband and parents. Approximately 1 μg of DNA was fragmented into 150–200 bp segments using a Covaris system, and exome capture was performed using the xGen Exome Research Panel v1.0 (IDT, USA). Sequencing was conducted on the MGISEQ‐2000 platform (BGI Genomics, China) after enrichment with the Roche KAPA HyperExome chip. Quality control ensured an average target region depth of ≥ 180×, with > 95% coverage at > 20×.

Sequencing reads were aligned to the hg19 reference genome using the Burrows‐Wheeler Alignment tool (BWA), with subsequent variant calling performed using GATK. Copy number variations (CNVs) were detected using ExomeDepth. Candidate pathogenic variants were filtered through databases including ClinVar, HGMD, and ClinGen, focusing on likely pathogenic variants (e.g., nonsense, frameshift, splice site mutations, etc.) with a population frequency < 1%. The Human Phenotype Ontology (HPO) database was used to identify variants related to the proband's phenotype. Sanger sequencing was conducted on the proband and parents to confirm and analyse variant segregation.

### 2.5 Pathogenicity Classification and Bioinformatic Prediction

The candidate variants were classified for pathogenicity following the guidelines of the American college of medical genetics and genomics (ACMG) [7]. *In silico* tools were utilised for predictive analysis of candidate variants' deleteriousness as follows.

Gene Model and Variant Localization: The structure of the CPLANE1 gene, consisting of 51 exons, was analysed using the Ensembl Genome Browser (version X). The positions of the c.203C>T and c.8893C>T variants were located in exons 3 and 48, respectively, and annotated using the genomic coordinates from the GRCh38 human genome assembly.

Mutation Hotspot Analysis: A mutation hotspot map for CPLANE1 was generated using publicly available databases, such as ClinVar and gnomAD, to identify and catalogue reported variants in the gene. A total of 3174 CPLANE1 variants were extracted, and the distribution of pathogenic variants was analysed across the gene's exons.

Amino Acid Conservation Analysis: The conservation of the 69th amino acid corresponding to the c.203C>T variant was evaluated across eight species (human, mouse, zebrafish, etc.) using the UCSC Genome Browser (version X) and multiple sequence alignment tools (Clustal Omega). Conservation was assessed to determine the evolutionary importance of the position.

Bioinformatics Prediction of Splicing and Protein Function: The potential impact of the c.203C>T variant on mRNA splicing and protein function was predicted using multiple bioinformatics tools. SpliceAI and Human Splicing Finder (HSF) were used to assess the likelihood of splicing alterations. SIFT, PolyPhen‐2, and MutationTaster were used to predict the functional consequences of the variant on protein stability and function.

RNA and DNA Damage Caused Splice Change (RDDC^SC^) Splicing Pattern Prediction: RDDC^SC^ was used to predict possible alternative splicing outcomes caused by the c.203C>T variant. The software analysed potential splice patterns, predicting three possible outcomes: (1) insertion of 81 bp leading to a premature stop codon, (2) deletion of 136 bp due to exon skipping causing a frameshift and premature stop codon, and (3) retention of the wild‐type splicing pattern.

### 2.6 In Vivo mRNA Splicing Validation

To investigate the effects of the identified variant c.203C>T in *CPLANE1* on mRNA alternative splicing, RNA was extracted from the normal control (wild‐type) and the mother's peripheral blood samples (carrying the variant c.203C>T) using the TRIzol reagent (Thermo Fisher Scientific, USA). Complementary DNA (cDNA) was synthesised from 1 μg of RNA using the PrimeScript RT Reagent Kit (Takara Bio, Japan), following the manufacturer's instructions. PCR amplification was performed using specific primers flanking the predicted splice sites. The PCR products were analysed by agarose gel electrophoresis to assess the presence of aberrant splicing events. Bands of interest were excised, purified, and sequenced using the Sanger method to confirm the precise splicing alterations.

### 2.7 Quantitative Real‐Time PCR (qRT‐PCR) for mRNA Expression

qRT‐PCR was performed using the SYBR Green PCR Master Mix (Applied Biosystems, USA) on a LightCycler 480 system (Roche, Switzerland). Specific primers were designed for the target gene and a reference housekeeping gene (e.g., GAPDH or β‐actin) for normalisation. The amplification protocol included an initial denaturation at 95°C for 10 min, followed by 40 cycles of 95°C for 15 s and 60°C for 1 min. All samples were run in triplicate. The relative expression levels were quantified using the 2^−ΔΔCt^ method, with the housekeeping gene serving as an internal control for normalisation.

### 2.8 Peripheral Blood Mononuclear Cell (PBMC) Isolation

Peripheral blood mononuclear cells (PBMCs) were isolated from anticoagulated whole blood using Ficoll‐Paque density gradient centrifugation. Whole blood was diluted 1:1 with sterile phosphate‐buffered saline (PBS) and carefully layered over Ficoll‐Paque in a 50 mL conical tube. The sample was centrifuged at 400–500 g for 30–40 min at room temperature without braking, resulting in the separation of plasma, PBMCs, Ficoll, and erythrocyte/granulocyte layers. The PBMC layer was carefully aspirated and transferred to a new tube, followed by washing with PBS and centrifugation at 300–400 g for 5–10 min. After 2–3 wash steps to remove contaminants, the PBMCs were resuspended in PBS or culture medium. Cell count and viability were assessed using a haemocytometer and trypan blue exclusion. PBMCs were then either used immediately for experiments or cryopreserved for later use.

### 2.9 Actinomycin D Assay for Assessing mRNA Stability in PBMCs

For the actinomycin D assay, PBMCs were cultured in RPMI 1640 medium supplemented with 10% fetal bovine serum (FBS) and antibiotics at 37°C with 5% CO_2_. After an initial recovery period of 24 h, actinomycin D was added to the culture at a final concentration of 5–10 μg/mL to inhibit transcription. Cells were harvested at different time points (e.g., 0, 1, 2, 4, and 6 h) following actinomycin D treatment. Total RNA was extracted from the harvested cells using TRIzol reagent, and RNA stability was assessed by quantitative PCR (qPCR) using specific primers for the target mRNA. The relative mRNA levels at each time point were calculated to determine the rate of mRNA decay and infer the mRNA stability.

### 2.10 Puromycin Treatment for Evaluating Translation‐Dependent mRNA Degradation in PBMCs

Puromycin treatment was performed to evaluate whether *CPLANE1* mRNA degradation in wild‐type (WT) and mutant (c.203C>T) PBMCs was translation‐dependent. Cells were cultured under standard conditions and treated with 10 μg/mL Puromycin for 4 h to inhibit protein translation. Following treatment, total RNA was extracted using TRIzol, and RNA quality was confirmed via Bioanalyzer (RIN > 7). Quantitative RT‐PCR (qRT‐PCR) was conducted to measure *CPLANE1* mRNA levels in both treated and untreated cells, using GAPDH as an internal control. The relative expression of *CPLANE1* mRNA was calculated using the 2^‐ΔΔCt method. The increase in *CPLANE1* mRNA levels in Puromycin‐treated mutant cells, compared to untreated controls, was analysed to assess the translation‐dependent degradation mechanism.

### 2.11 SMG1 Inhibitor Treatment and Cell Viability Assay Using CCK‐8

PBMCs from both wild‐type (WT) and mutant (c.203C>T) groups were cultured in RPMI 1640 medium supplemented with 10% fetal bovine serum (FBS) and 1% penicillin–streptomycin at 37°C with 5% CO_2_. After an initial recovery period of 24 h, the cells were treated with different concentrations of the SMG1 inhibitor GSK2830371 (0.1 μM, 0.5 μM, 1 μM, and 5 μM) for a total duration of 24 h. Control groups were treated with DMSO (vehicle control).

Cell viability was assessed at 0, 6, 12, 18, and 24 h post‐treatment using the Cell Counting Kit‐8 (CCK‐8) assay (Dojindo). 10 μL of CCK‐8 solution was added to each well, followed by incubation at 37°C for 2 h. The absorbance was measured at 450 nm using a microplate reader. Cell viability was expressed as a percentage relative to the DMSO control. The effect of SMG1 inhibition on cell viability was analysed by comparing the viability of WT and mutant cells across the different concentrations of GSK2830371.

### 2.12 SMG1 Inhibitor Treatment and Quantification of *CPLANE1* mRNA Expression Using qRT‐PCR for Evaluating the Involvement of the NMD Pathway

To assess the impact of SMG1 inhibition on *CPLANE1* mRNA expression, PBMCs from both wild‐type (WT) and mutant (c.203C>T) groups were cultured in RPMI 1640 medium with 10% FBS and 1% penicillin–streptomycin at 37°C with 5% CO_2_. After 24 h of recovery, cells were treated with 1 μM GSK2830371 for 12 h. Untreated cells served as controls.

Total RNA was extracted from treated and control cells using the TRIzol reagent (Invitrogen). RNA was reverse transcribed into cDNA using a reverse transcription kit (Thermo Fisher Scientific), followed by quantitative RT‐PCR (qRT‐PCR) to measure *CPLANE1* mRNA levels. GAPDH was used as an internal control, and the relative expression of *CPLANE1* mRNA was calculated using the 2^‐ΔΔCt method. The effect of SMG1 inhibition on *CPLANE1* mRNA expression was analysed by comparing the expression levels in treated WT and mutant cells with their respective untreated controls.

### 2.13 Statistical Analysis

All experiments were performed in triplicate, and data were presented as mean ± standard deviation (SD). Statistical analysis was conducted using GraphPad Prism (version 9). Differences between groups were evaluated using an unpaired two‐tailed Student's t‐test for comparisons between two groups, and one‐way ANOVA followed by Tukey's post hoc test for comparisons among multiple groups. For cell viability and mRNA expression data, statistical significance was set at *p* < 0.05.

## 3 Results

### 3.1 Imaging and Pathology Confirm Multiple Abnormalities

**FIGURE 2:**
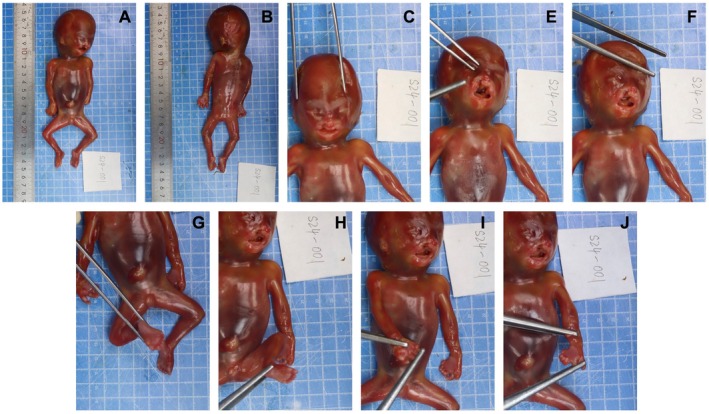
Morphological abnormalities observed in the fetus terminated at 17 weeks and 3 days of gestation from the third adverse pregnancy. (A, B) Overview of the fetus showing general morphology and scale. (C, D) Detailed views highlighting bilateral cleft lip and cleft palate (C), flat nasal bridge (D), and overall facial dysmorphology. (E, F) Close‐up images showing the severity of the craniofacial anomalies, including the extensive clefting of the upper lip and palate. (G, H) Images depicting the lower limbs with evident bilateral clubfoot and toe syndactyly. (I, J) Images focusing on the upper limbs, illustrating syndactyly of the fingers, consistent with complex limb malformations. All images were taken during a detailed post‐mortem examination to document the phenotypic manifestations of the genetic condition.

1. Prenatal Ultrasound at 26 Weeks of Gestation during the second pregnancy (Figure 1): In the second adverse pregnancy, a 26‐week ultrasound revealed several abnormalities: absence of the cerebellar vermis with communication between the cisterna magna and the fourth ventricle, a 0.65 cm echogenic discontinuity on the right side of the upper lip, nasal wing depression, a 0.3 cm discontinuity in the alveolar ridge, and a 0.18 cm discontinuity in the palate. Additionally, the fetal hands exhibited abnormal positioning, with possible syndactyly, and a polydactyly‐like structure was noted on the lateral aspect of both fifth toes.
2. Prenatal Ultrasound at 17 + 3 Weeks of Gestation during the third pregnancy: Prenatal ultrasound revealed bilateral interruptions of the upper lip, each measuring 0.17 cm, with the absence of normal nasal echoes. A nasal‐like structure measuring 0.55 × 0.35 cm was noted above the upper lip. The third ventricle had an inner diameter of 0.36 cm, while both lateral ventricles measured 1.0 cm. Cerebellar malformation was identified, along with an enhanced intestinal echo measuring approximately 2.7 × 2.5 cm.
3. Pathological Findings of the Proband (Figure 2): The fetus exhibited bilateral cleft lip and palate (including cleft lip, alveolar ridge cleft, and cleft palate), nasal malformation, seven fingers with syndactyly on both hands, and seven toes with syndactyly on both feet. There was partial absence of the cerebellar vermis, with a reduction in surrounding neurons and mild proliferation of nerve fibres. Bilateral lateral ventricles were dilated.

### 3.2 WES and Sanger Sequencing Identify Novel Compound Heterozygous Variants in *CPLANE1*

**FIGURE 3:**
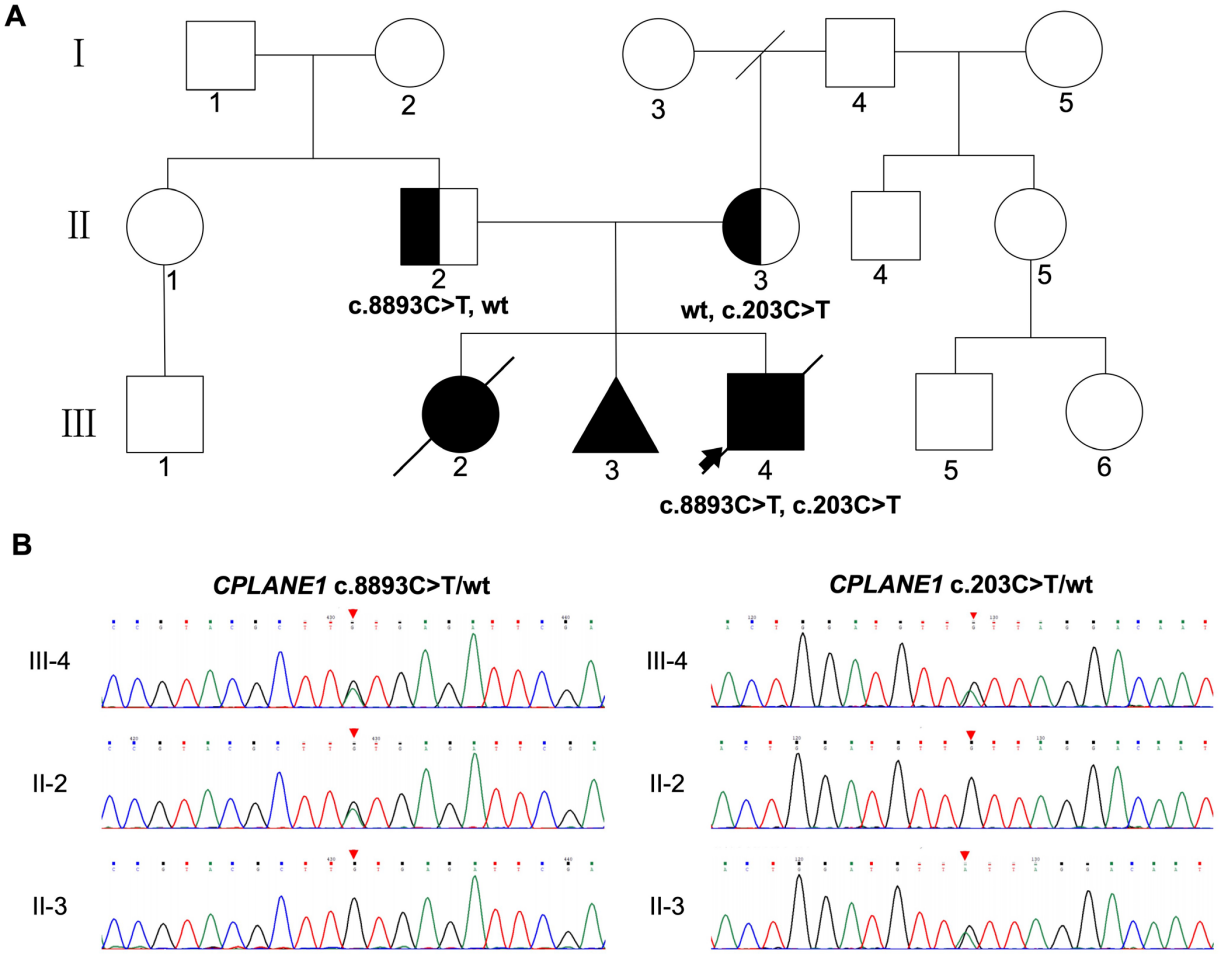
Pedigree and Sanger sequencing results of a family with compound heterozygous variants in *CPLANE1* associated with Joubert syndrome. (A) The family pedigree shows the segregation of two compound heterozygous variants in *CPLANE1*: C.8893C>T (p.Gln2965*) and c.203C>T (p.Thr68Ile). Affected individuals are represented by filled symbols, and the proband (III‐4) is marked with an arrow. Individual II‐2 carries the c.8893C>T variant, while individual II‐3 carries the c.203C>T variant, both in heterozygous form. (B) Sanger sequencing chromatograms for the c.8893C>T and c.203C>T variants in the proband (III‐4) and parents (II‐2 and II‐3). The proband inherited both variants in a compound heterozygous state, while the parents are each heterozygous carriers for one of the variants.

WES analysis of the proband's peripheral blood DNA showed 99.99% coverage of target genes, with an average sequencing depth of 259.18×. Analysis of the ClinVar, HGMD, ClinGen, and HPO databases identified compound heterozygous variants in the *CPLANE1* gene (OMIM 600415) on chromosome 5, associated with the proband's clinical phenotype. The identified variants were novel c.8893C>T (p.Gln296*5*) and c.203C>T (p.Thr68Ile). Sanger sequencing confirmed that the father carried the c.8893C>T (p.Gln2965*) variant in a heterozygous state, while the mother carried the c.203C>T (p.Thr68Ile) variant in a heterozygous state (Figure 3). *CPLANE1* gene variants are associated with Joubert syndrome type 17 (OMIM:614615) and oral‐facial‐digital syndrome type 6 (OMIM:277170), both inherited in an autosomal recessive (AR) pattern.

### 3.3 Pathogenicity Analysis Reveals Likely Pathogenic and VUS Variants in *CPLANE1*

Database searches of gnomAD, dbSNP, ExAC, ESP, and 1000 Genomes revealed that the *CPLANE1* gene c.8893C>T (p.Gln2965*) variant was located in exon 48, resulting in a C‐to‐T substitution at nucleotide position 8893, leading to a nonsense mutation. This mutation introduces a premature stop codon at amino acid position 2965 (glutamine), potentially triggering nonsense‐mediated mRNA decay. Loss‐of‐function mutations in CPLANE1 have been reported as pathogenic (PVS1), and this variant has not been previously reported in the literature or databases (ExAC, ESP, 1000 Genomes, gnomAD) (PM2). Based on ACMG guidelines, this variant is classified as likely pathogenic (PVS1 + PM2).

The *CPLANE1* gene c.203C>T (p.Thr68Ile) variant was located in exon 3, causing a missense mutation that replaces threonine with isoleucine at position 68. This variant has not been previously reported in the literature or in public databases (ExAC, ESP, 1000 Genomes, gnomAD) (PM2). In autosomal recessive inheritance, the presence of a pathogenic variant in trans with another pathogenic variant has been observed (PM3). According to ACMG guidelines, this variant is classified as a variant of uncertain significance (VUS) (PM2 + PM3).

### 3.4 Bioinformatics Predictions of c.203C>T (p.Thr68Ile) in *CPLANE1*

Given the proband's maternal history of three similar adverse pregnancies, with carrying novel compound heterozygous variants in *CPLANE1*, these variants were strongly suspected to be the underlying cause of fetal Joubert syndrome. However, based on the ACMG guidelines, the current evidence only supports the classification of *c.203C>T* as a VUS. To further evaluate its pathogenicity and provide accurate genetic counselling and reproductive strategies such as PGT‐M, we conducted bioinformatics analyses to predict the deleteriousness of *c.203C>T*, assessing its potential impact at both the mRNA splicing and protein translation levels.

**FIGURE 4:**
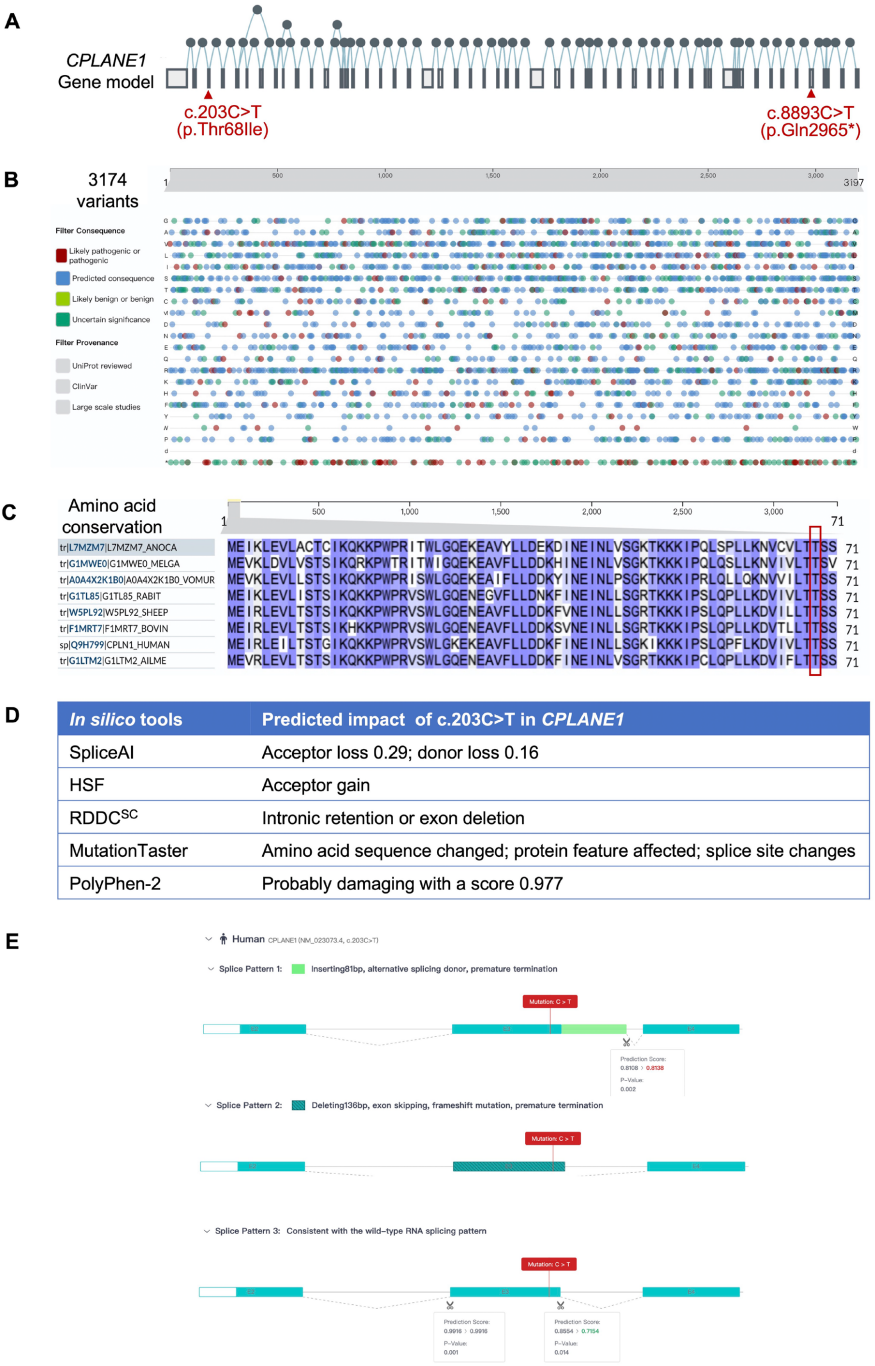
Bioinformatic analysis of c.203C>T and c.8893C>T in *CPLANE1*. (A) The gene model shows that *CPLANE1* consists of 51 exons, with the c.203C>T variant located in exon 3 and the c.8893C>T variant located in exon 48. (B) The mutation hotspot map indicates that 3174 variants in *CPLANE1* have been reported in the database, with pathogenic variants evenly distributed. (C) Amino acid conservation analysis reveals that the 69th amino acid position, corresponding to the c.203C>T variant, is highly conserved among eight species. (D) Various online bioinformatics tools predict the impact of the c.203C>T variant on mRNA splicing and protein function. (E) RNA and DNA Damage Caused Splice Change (RDDC^SC^) predicts that the c.203C>T variant could lead to three splice patterns: Pattern 1 involves the insertion of 81 bp, resulting in a premature stop codon; pattern 2 involves the deletion of 136 bp due to exon skipping, leading to a frameshift and premature stop codon; pattern 3 corresponds to the wild‐type (WT).

The gene model revealed that these variants are located in exons 3 and 48, respectively, within functionally significant regions of *CPLANE1* (Figure 4A). A mutation hotspot analysis (Figure 4B) identified 3174 reported variants across the gene, with pathogenic variants distributed throughout, highlighting the importance of these exonic regions. Conservation analysis showed that the 69th amino acid, corresponding to the c.203C>T variant, is highly conserved across species (Figure 4C), suggesting that changes at this position may impair protein function. Predictions from multiple in silico tools indicated that the c.203C>T variant could disrupt splicing by either inducing intronic retention or exon deletion, leading to frameshift mutations and premature termination (Figure 4D). RDDC^SC^ further predicted three possible splicing patterns for this variant, two of which result in abnormal splicing and likely loss of function (Figure 4E).

In summary, bioinformatics predictions suggest that the *c.203C>T* (*p.Thr68Ile*) variant likely contributes to the pathogenicity of Joubert syndrome by affecting both mRNA splicing and protein function.

### 3.5 C.203C>T in *CPLANE1* Causes Aberrant Alternative Splicing and Frameshift In Vivo

**FIGURE 5:**
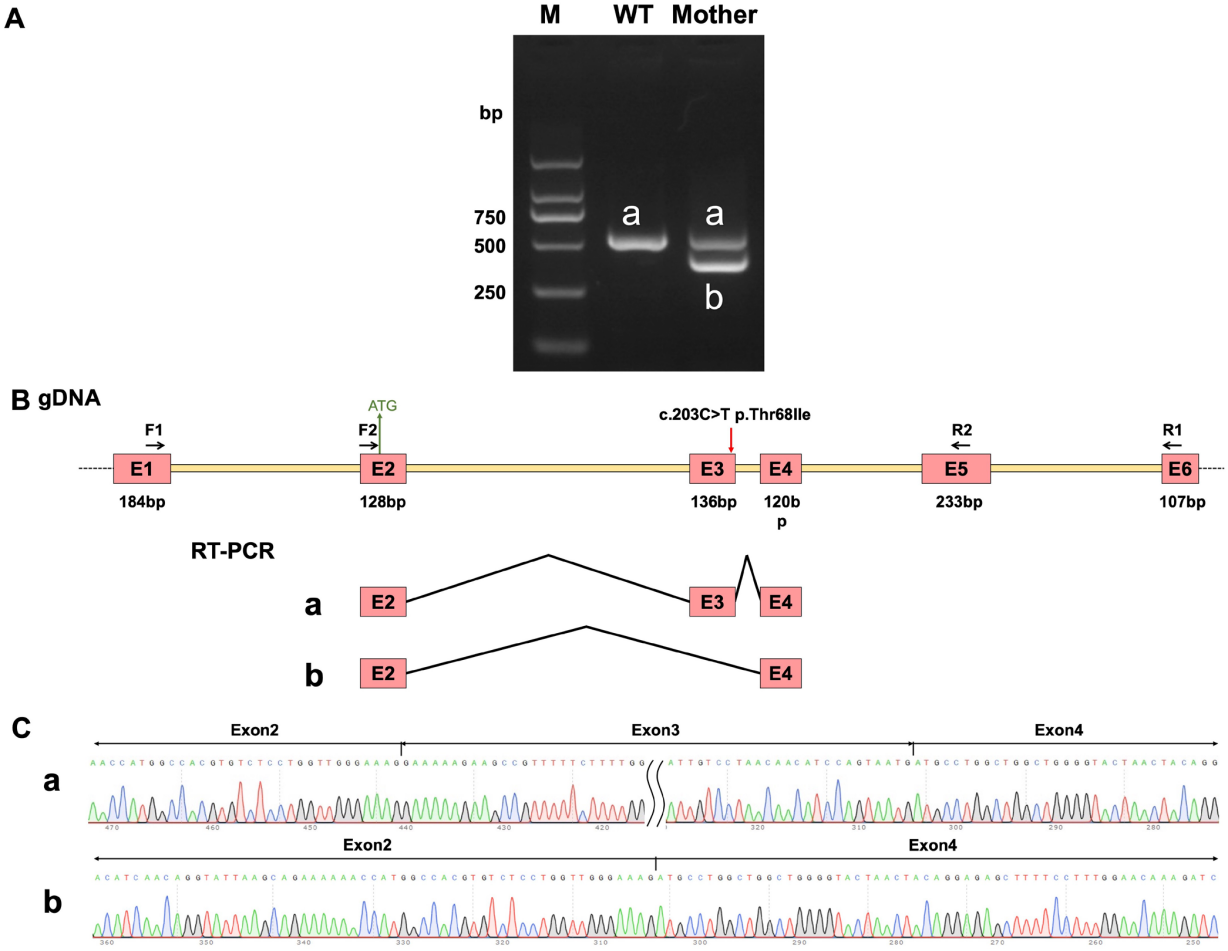
In vivo assay to assess the effect of the c.203C>T variant in *CPLANE1* on mRNA splicing. (A) Agarose gel electrophoresis of RT‐PCR products. Band ‘a’ represents the normally spliced product observed in the wild‐type control (WT). In the proband's mother, two bands are detected: Band ‘a,’ corresponding to the wild‐type splicing pattern, and band ‘b,’ representing an aberrant splicing product. (B) Schematic representation of primer design and splicing events. The red arrow indicates the c.203C>T variant. RT‐PCR reveals two splicing forms: One corresponding to normal splicing and another resulting from the skipping of exon 3. (C) Sequencing chromatograms of the RT‐PCR products. Band ‘a’ corresponds to the normal splicing event, including exons 2, 3, and 4, while band ‘b’ shows exon 3 skipping, resulting in the direct joining of exons 2 and 4.

To evaluate the effect of the c.203C>T variant on gene transcription, RT‐PCR was performed using cDNA from the proband's mother and normal control as templates. The results showed a single band in the control's sample (designated as band a). Sanger sequencing confirmed that this band represented the normal transcript, with the splicing pattern of Exon2 (128 bp)—Exon3 (136 bp)—Exon4 (120 bp), indicating that the control does not carry the c.203C>T (p.Thr68Ile) variant and thus only produces a normal splice product. In the mother's sample, two bands were observed (designated as band a and band b) (Figure 5A). Band a corresponded to the normal splicing product with the same pattern as the control's (WT's), while band b was identified as an aberrant transcript, showing exon 3 skipping, which resulted in the direct joining of Exon2 (128 bp) and Exon4 (120 bp) (Figure 5B). Further Sanger sequencing analysis confirmed that the mother is a heterozygous carrier of the c.203C>T (p.Thr68Ile) variant, leading to the production of both normal and aberrant transcripts (Figure 5C). The exon 3 skipping caused by the variant leads to a frameshift, creating a premature termination codon (PTC) within Exon4, potentially producing a truncated protein of 34 amino acids. This abnormal splicing event is represented at the cDNA and protein levels as c.82_217del (p.Glu28Metfs*8).

### 3.6 C.203C>T in *CPLANE1* Triggers Nonsense‐Mediated mRNA Decay

**FIGURE 6:**
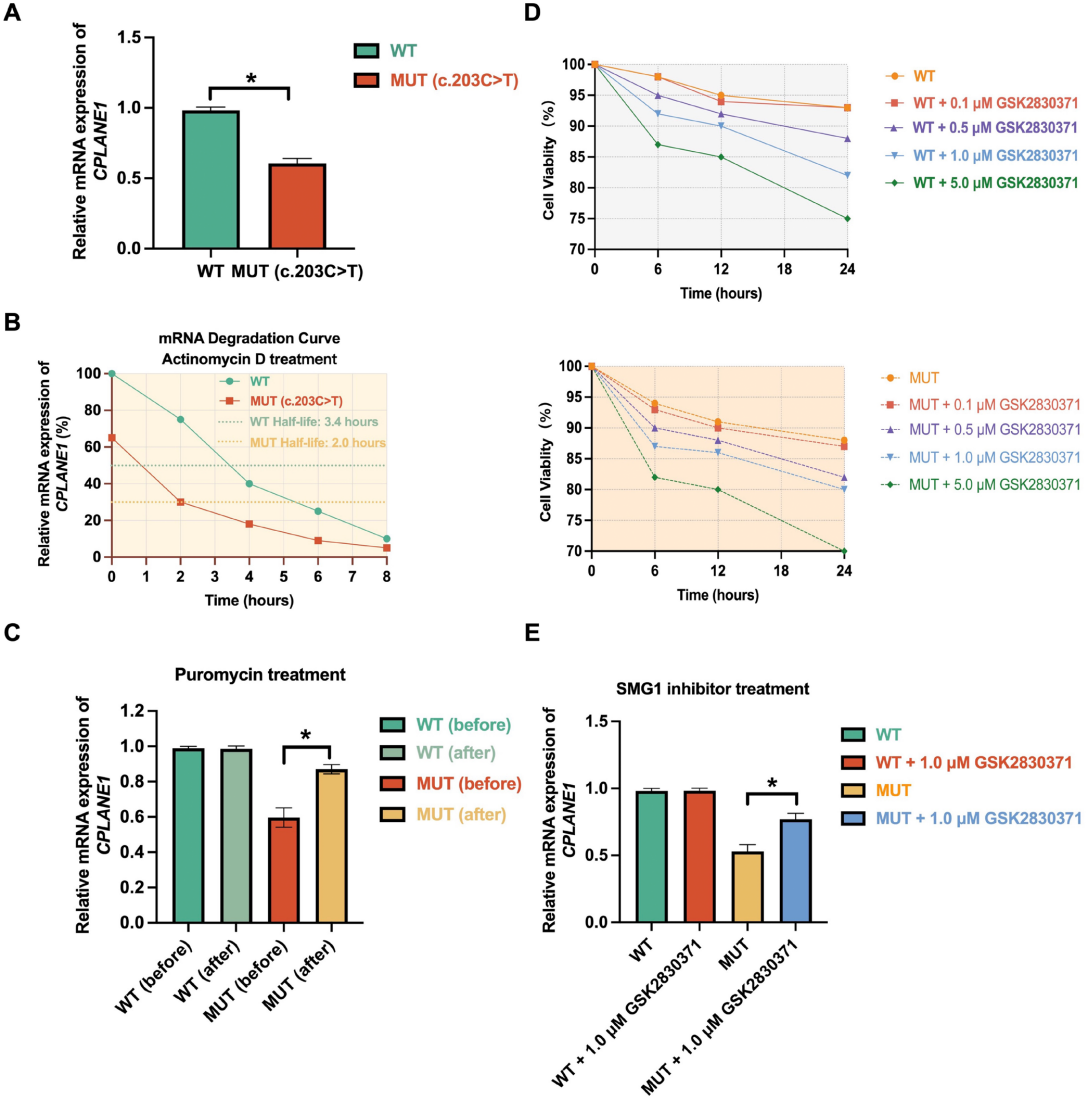
c.203C>T in *CPLANE1* Triggers nonsense‐mediated mRNA decay. (A) Relative mRNA expression of *CPLANE1* in Wild Type (WT) and Mutant Type (MUT, c.203C>T) cells. The expression of *CPLANE1* is significantly reduced in the mutant group compared to the wild‐type. (B) mRNA degradation curve following Actinomycin D treatment for WT and MUT cells. The half‐life of *CPLANE1* mRNA is shorter in the mutant group (2.0 h) compared to the wild‐type (3.4 h), indicating accelerated mRNA decay in the mutant. (C) Relative *CPLANE1* mRNA expression before and after Puromycin treatment. In the mutant group, Puromycin treatment leads to a significant increase in mRNA expression, suggesting that the mRNA is degraded by a translation‐dependent mechanism. (D) PBMC cell viability in WT and Mutant cells treated with different concentrations of SMG1 inhibitor (GSK2830371) over 24 h. Both WT and Mutant cells show a dose‐dependent reduction in cell viability, with higher concentrations (5 μM) causing a more pronounced decrease in viability. (E) Relative *CPLANE1* mRNA expression in WT and Mutant cells treated with 1.0 μM SMG1 inhibitor (GSK2830371) after 12 h. The inhibitor significantly increases mRNA expression in the mutant group, suggesting NMD pathway involvement in mRNA degradation *indicates *p* < 0.05.

To investigate the effect of the c.203C>T mutation on *CPLANE1* mRNA expression, we performed quantitative RT‐PCR (qRT‐PCR) to compare the relative mRNA levels between wild‐type (WT) and mutant (MUT) cells. As shown in Figure 6A, *CPLANE1* mRNA expression was significantly reduced in the mutant group compared to the WT group (*p* < 0.05), indicating that the c.203C>T mutation leads to a reduction in mRNA stability or expression.

We next examined whether the reduced mRNA expression in mutant cells was due to accelerated mRNA degradation. To this end, we treated both WT and mutant cells with Actinomycin D to inhibit transcription and measured *CPLANE1* mRNA levels over time. As shown in Figure 6B, the half‐life of *CPLANE1* mRNA in mutant cells was significantly shorter (2.0 h) compared to WT cells (3.4 h). This result indicates that the c.203C>T mutation accelerates the degradation of *CPLANE1* mRNA.

To determine whether the c.203C>T mutation leads to translation‐dependent degradation of *CPLANE1* mRNA, we treated WT and mutant cells with Puromycin, a translation inhibitor. Puromycin treatment resulted in a significant increase in *CPLANE1* mRNA levels in mutant cells (*p* < 0.05), whereas no significant change was observed in WT cells (Figure 6C). This suggests that the mutant *CPLANE1* mRNA undergoes degradation via a translation‐dependent mechanism, possibly through Nonsense‐Mediated Decay (NMD).

We further assessed the impact of SMG1 inhibition on cell viability using the SMG1 inhibitor GSK2830371 at different concentrations. PBMCs from both WT and mutant groups were treated with increasing concentrations of the inhibitor (0.1 μM, 0.5 μM, 1.0 μM, 5.0 μM) over a period of 24 h. A dose‐dependent reduction in cell viability was observed in both WT and mutant cells, with higher concentrations (5.0 μM) showing a more pronounced decrease in viability (Figure 6D). Therefore, we selected a treatment condition of 1 μM SMG1 inhibitor for 12 h to assess mRNA expression in subsequent experiments.

Results displayed that the inhibition of SMG1 significantly increased *CPLANE1* mRNA levels in mutant cells (*p* < 0.05), while no significant change was observed in WT cells (Figure 6E). This suggests that the degradation of mutant *CPLANE1* mRNA is mediated by the NMD pathway, which is regulated by SMG1 activity.

Taken together, these results demonstrate that the c.203C>T variant in *CPLANE1* causes exon skipping and a frameshift, introducing a premature termination codon (PTC) that triggers nonsense‐mediated mRNA decay (NMD), a mechanism that degrades mRNAs containing PTC.

According to ACMG guidelines, the c.203C>T variant can be upgraded from VUS to Likely Pathogenic (LP) based on multiple lines of evidence. PS3: Functional studies demonstrate that the variant causes exon skipping, leading to abnormal mRNA degradation via NMD, providing strong evidence of disrupted gene function. PVS1: The variant results in a premature stop codon, consistent with a loss‐of‐function mechanism known to cause disease in *CPLANE1*. PP3: Computational tools predict significant splicing and protein disruption. PM2: The variant is absent from population databases, further supporting its pathogenicity. Together, these factors meet the criteria for Likely Pathogenic.

## 4 Discussion

In this study, we identified novel compound heterozygous variants in *CPLANE1*, c.8893C>T (p.Gln2965*) and c.203C>T (p.Thr68Ile), in a family with recurrent fetal abnormalities indicative of Joubert syndrome (JS). The c.8893C>T variant, a nonsense mutation, was classified as likely pathogenic due to its predicted loss‐of‐function through NMD, consistent with known *CPLANE1* loss‐of‐function mutations associated with JS [8].

The c.203C>T variant was initially classified as VUS, but bioinformatics predictions indicated that this variant likely affects both mRNA splicing and protein function. In vivo splicing assays confirmed that the c.203C>T variant results in exon 3 skipping, leading to a frameshift mutation and the production of a truncated protein. The generation of a premature stop codon in exon 4 triggered NMD, reducing the stability and expression of the mutated mRNA. These functional consequences strongly suggest that the c.203C>T variant plays a pathogenic role in JS, although further studies may be required to fully elucidate its impact on ciliogenesis and neural development.

JS is a ciliopathy characterised by significant genetic heterogeneity, typically associated with the “molar tooth sign” on MRI [9]. In this family, however, the foetuses presented with atypical prenatal findings, including cleft lip, ventriculomegaly, cerebellar vermis hypoplasia, and polydactyly, but lacked the characteristic “molar tooth sign (MTS)”. This variability could reflect the nonspecific presentation of JS in early gestation or limitations in prenatal imaging techniques. This atypical presentation could be attributed to several factors: Timing and Resolution of Imaging, Genetic Modifiers, Limitations in Prenatal Imaging Techniques.

Firstly, prenatal imaging techniques, such as MRI, may have limitations in resolution, especially if performed at an early gestational age [10]. The timing of imaging is crucial, as imaging performed too early might not capture the full development of brain structures [11]. Additionally, the resolution of prenatal MRI may not be sufficient to detect the MTS in all cases, particularly in foetuses with smaller brain structures [12]. Secondly, phenotypic variability in JS can also be influenced by genetic modifiers. Variants in other genes, in addition to the primary causal mutations, may affect the severity and presentation of the disease [13]. This suggests that the absence of the MTS in some family members could be due to the presence of additional genetic factors that modify the phenotypic expression of JS. Thirdly, the limitations of prenatal imaging techniques, such as ultrasound and MRI, can impact the accuracy of early JS diagnosis. Ultrasound techniques for analysing fetal growth patterns may not always detect subtle brain anomalies, and MRI resolution can vary depending on the gestational age and imaging protocols [14].

The phenotypic variability observed in our study highlights the importance of a comprehensive diagnostic approach for JS. Comprehensive imaging, including fetal MRI, is crucial for improving the diagnostic accuracy of JS in high‐risk pregnancies [15]. While imaging is a critical tool, it should be complemented with genetic testing, such as whole‐exome sequencing and Sanger sequencing, to identify the underlying genetic mutations. This integrated approach can help in providing a more accurate diagnosis, especially in cases where imaging results are inconclusive or atypical. Moreover, the identification of novel mutations in the *CPLANE1* gene, as demonstrated in our study, further expands the mutational spectrum of JS and underscores the need for continued research into the genetic basis of the disease. This will not only enhance our understanding of JS but also improve the accuracy of genetic counselling and diagnosis.

To date, more than 35 genes have been implicated in JS, with *CPLANE1* being the 17th identified pathogenic gene. *CPLANE1* mutations cause JS type 17, which manifests with cerebellar ataxia, developmental delay, and polydactyly [16]. Consistent with previous findings, the three affected foetuses in this family exhibited polydactyly, cerebellar hypoplasia, and cleft lip/palate, indicating incomplete phenotypic expression of JS. Whole‐exome sequencing (WES) and Sanger sequencing confirmed the compound heterozygous *CPLANE1* mutations, with c.8893C>T classified as likely pathogenic, and c.203C>T classified as a VUS. These variants are novel and have not been previously reported, expanding the mutational spectrum of *CPLANE1* in JS.

*CPLANE1* (NCBI Gene ID: 65250) encodes a protein involved in ciliogenesis and planar cell polarity. It is also known as *Hug*, *OFD6*, *JBTS17*, and *C5orf42*, and is located on chromosome 5p13.2, comprising 52 exons and encoding a protein of 3197 amino acids. *CPLANE1* is expressed in various tissues and plays a role in establishing the polarity necessary for directed cell movement, although its exact function in JS is still unclear [17]. Silencing *CPLANE1* in chicken embryos disrupted axon formation in both the central and peripheral nervous systems and affected craniofacial development [18]. Hong et al. demonstrated that *CPLANE1* is crucial for chromosome alignment and spindle pole positioning during mitosis, and its deficiency in a mouse model affected mitotic progression in cortical neural progenitors, impairing post‐mitotic neuronal migration [19]. Toriyama et al. found that knockdown of *CPLANE1* in Xenopus embryos caused ciliopathy‐related developmental defects, including neural tube closure failure, abnormal Hedgehog signalling, and left–right patterning defects [20]. In mouse studies, *CPLANE1* knockdown specifically interfered with the recruitment of peripheral intraflagellar transport protein (IFT) subunits to the basal body, affecting ciliogenesis [20].

The c.8893C>T nonsense mutation in *CPLANE1* is located in exon 48 and results in a stop codon at amino acid position 2965, leading to a truncated protein. This variant is highly conserved across species, indicating its crucial role in maintaining *CPLANE1* function. The c.203C>T (p.Thr68Ile) missense mutation in exon 3 changes threonine to isoleucine at position 68. Although this variant has not been reported in databases such as ExAC or gnomAD, in vivo experiments showed that it causes exon 3 skipping, a frameshift, and premature stop codon formation, triggering NMD. Together, bioinformatic analysis combined with experimental results supports the reclassification of this variant from VUS to LP, providing important guidance for clinical genetic counselling.

Given that the *CPLANE1* compound heterozygous mutations were inherited from both parents, there is a 25% risk of recurrence in future pregnancies. According to the “Preimplantation Genetic Testing: ACOG Committee Opinion”, PGT is recommended for this couple to ensure the birth of healthy offspring [21]. Alternatively, close monitoring of fetal development during future pregnancies, coupled with invasive prenatal diagnostic testing, is advised to determine whether the fetus carries pathogenic variants.

In summary, we identified novel pathogenic *CPLANE1* variants through WES and bioinformatic analyses, followed by confirmation via Sanger sequencing and in vivo experiments. This approach allowed us to determine that the c.8893C>T and c.203C>T compound heterozygous variants likely caused JS in the proband. By reviewing the clinical characteristics and genetic data of this family, we aim to raise awareness of JS among clinicians to prevent misdiagnosis or missed diagnoses. Our findings expand the mutational spectrum of JS, elucidate its molecular mechanisms, and provide valuable insights for clinical diagnosis, genetic counselling, and reproductive guidance.

## Author Contributions

**Mei Wang:** conceptualization (lead), data curation (lead), formal analysis (lead), funding acquisition (lead), investigation (equal), methodology (equal), project administration (equal), resources (equal), software (equal), supervision (equal), validation (equal), visualization (equal), writing – original draft (lead), writing – review and editing (equal). **Zhidan Hong:** conceptualization (lead), data curation (supporting), project administration (equal), writing – original draft (equal). **Sheng Xiang:** formal analysis (equal), investigation (equal), writing – review and editing (equal). **Zhiying Chen:** data curation (equal), investigation (equal), resources (equal), validation (equal). **Xueping Qiu:** resources (equal), writing – review and editing (equal). **Li Zhang:** resources (equal). **Ling Ma:** conceptualization (equal), investigation (equal), project administration (equal), resources (equal), supervision (equal).

## Ethics Statement

All procedures were in accordance with the Declaration of Helsinki. The research protocols were approved by the Ethics Committee of Zhongnan Hospital, Wuhan University, Wuhan, China (Ethics No. 2023068K).

## Conflicts of Interest

The authors declare no conflicts of interest.

## Data Availability

The datasets generated during the current study are available from the corresponding author on reasonable request.
